# Are season of birth, climate, and parental age predictors of adult temperament, character, anhedonia, suicidality, and self-harm?

**DOI:** 10.3389/fpsyt.2025.1710173

**Published:** 2025-11-06

**Authors:** Konstantinos N. Fountoulakis, Nikolaos K. Fountoulakis

**Affiliations:** 3rd Department of Psychiatry, School of Medicine, Aristotle University of Thessaloniki, Thessaloniki, Greece

**Keywords:** temperament, character, season of birth, parental age, suicidality

## Abstract

**Introduction:**

The aim of the current study was to investigate the relationship of temperament and suicidality with the season of birth, climate variables, and parental age.

**Material and methods:**

The study sample included 701 subjects (aged 18–67 years; 59.48% females). The protocol gathered the precise date of birth, parental age at delivery, temperament and personality data (TEMPS-A, NEO-PI-3, TCI, and CAMT), and suicidality data (RASS). Epidemiological data concerning suicides and climate data were also obtained.

**Results:**

None of the ‘classic’ temperament and personality variables of the three major models (TEMPS, TCI, and NEO-PI) manifest any relationship with season of birth, climatic variables at birth, or parental age. Only the Internalized Interpersonal Emotion (IIE) from the CAMT manifested high scores for those born during spring and low scores for those born in summer and autumn. There was a weak but significant correlation between weight and BMI with all climate variables. Deaths by suicide manifested higher rates during the spring. Zodiacs were not related to any temperament or character facet.

**Discussion:**

Overall, our results point to the importance of the interplay between environmental and biological determinants of temperament and suggest that spring is related to deficits in affective interpersonal relating for those born during spring, and also to higher deaths by suicide in adults again during the same season. This could mean that this period of the year exerts an adverse effect on mental health, at least on vulnerable populations. Future research should focus on neurobiological differences in relation to the season of birth and climate, especially in the face of the changes in mental health that climate change might cause.

## Introduction

The season of birth used to be an important element in the pre-scientific era of medicine (1, 2). In this frame of the beliefs held since ancient times and until relatively recently, the border between humans and the universe could be considered arbitrary, and man was believed to follow the laws of nature, in a sense radically different from what is accepted today. The remains of these beliefs had dominated psychiatry and psychology for more than 2000 years, and today they survive in the lay people’s beliefs about astrology and zodiacs. On the other hand, the modern theories concerning the etiopathogenesis of mental disorders (according to the biopsychosocial model) include the influence of environmental factors (including climate), especially during gestation or the first months and years of life.

Recently, large-scale population studies on millions of people reported associations between neurological, respiratory, cardiovascular, and reproductive conditions (3–5), type I diabetes (6), weight and body size in childhood (7), as well as seasonal affective and bipolar disorder and schizophrenia (8), with birth month and season. However, no seasonal birth month effect was observed for overall mortality, at least not among women (3). There is evidence that children born during the first months of the year might achieve higher academic performance (9) and that the season of birth constitutes a determinant of emotional and behavioral maturation in adult life (10). Apart from mental disorders, being born during spring might predispose one to death by suicide in adulthood (11), while being born during the autumn is related to a preference for hanging as the method of choice (12). Variables like the environmental light have been shown to have a significant effect on body function (13).

Temperament and personality theories were always influencing philosophical thinking. They played a predominant role in the shaping of the anthropological and humanitarian sciences, since ancient Egypt and Mesopotamia, and the development of the theory of bodily humors in ancient Greece by the school of Cos and specifically by Polybos, a pupil and son-in-law to Hippocrates (4th century B.C). Temperament originally referred to those aspects of personality that are innate rather than learned, whereas character was considered to be what we made of ourselves intentionally. Later, it was considered that temperament is the emotional core of personality that is moderately stable throughout life, whereas character reflects a person’s goals and values as they develop over the lifespan (14, 15).

Currently, the theories of temperament and character are represented by three major questionnaires, the NEO-PI-3 (the Big Five personality traits) (16–18), the TCI (19–22), and the TEMPS-A (23–31). However, these are not the only ones that exist (32), and a recent cognitive-affective model (CAMT) has been proposed (33, 34). The first three correspond to three dominant models of temperament and personality today, and there exist significant theoretical and also essential differences between them. McCrae and Costa proposed the five-factor model (Big Five) (35), which includes neuroticism, extroversion, agreeableness, openness, and conscientiousness and constitutes a further development of Eysenck’s theory. The older concept of ‘psychoticism’ was substituted by agreeableness and conscientiousness, while openness has some degree of overlap with extroversion (36). Their work is primarily based on the classical psycholexical study by Gordon Allport and Henry Odbert (37, 38) and produced the NEO-PI-3. The work of Robert Cloninger is characterized by an attempt to intimately connect temperamental characteristics with individual differences in genetics, neurotransmitter systems, and behavioral conditioning. He described novelty seeking (anger), harm avoidance (fear), reward dependence (attachment), and persistence (ambition) (19, 22). His research suggests that temperament components can be assessed as early as preschool age (20) and remain moderately stable throughout a person’s lifespan except for changes from behavioral conditioning (39). Cloninger’s model is reflected in the TCI. Hagop Akiskal conceived temperament as the affective predisposition or reactivity, based on the original descriptions of fundamental states (manic or hyperthymic, irritable, cyclothymic, anxious, and depressive) by Kraepelin in 1921. This model concerns exclusively the affective temperament modules and has been conceived while evaluating and observing mood patterns in clinical practice (23, 40–42). It is reflected in the TEMPS-A.

The combination of the above models gave rise to a novel proposal, a cognitive-affective model of temperament (CAMT) with 12 characters at the base which include Ego Resiliency (ER), Ego Strength (ES), Intrapersonal Emotion (IE), Personal Space Cognition (PSC), Interpersonal Cognition (IC), Emotional Creativity (EC), Externalized Interpersonal Emotion (EIE), Internalized Interpersonal Emotion (IIE), Emotional Motivation (EM), Self-Discipline (SD), Ethical Values (EV), and Ethical Behavior (EB), six higher level temperaments corresponding to Emotional Self (EmoS), Cognitive Self (CogS), Social Emotionality (SE), Emotional and Cognitive Control (ECC), Ethical Emotionality and Behavior (EEB), Social Emotionality and Behavior (SEB) and two super-factors at the top, the Self (S) and the Self-Environment Interactions (SEI). For a complete description of this model, the reader should seek the original publications (33, 34). The gross structure of this model suggests that at the core of psychological function are the internal emotional and cognitive processes, which through social emotionality and meta-cognition determine the externalized behavior, which is further shaped by internalized social factors in the form of ethical values. Interestingly, both meta-cognitive modules (ECC and EEB) are not purely cognitive, but they include a strong emotional component (EM and IIE).

So far, empirical research on the developmental parameters of temperament and personality is rather limited, even after taking into consideration the enormous efforts by generations of researchers. Especially limited are the available data concerning the effect of environmental factors during gestation and the early months of life, on temperament and personality. Especially little is known on the possible effect of climate on them.

The aim of the current study was to investigate the relationship between anxiety, distress, depressive symptoms (not depression), temperament, and character, as well as of suicidal thoughts and self-harm at present, with season of birth, climate variables at delivery, and parental age at delivery. A secondary aim was to investigate whether the seasonality of suicides is related to any seasonality in the relationship between birth and temperament and character.

## Materials and methods

### Material

The study sample included 701 subjects from the general Greek population from a dataset of 734 subjects who participated in the standardization of temperament and character scales and the development of the CAMT model of temperament and personality (33, 34, 43–48). Complete data for the aims of the current study were available for these 701 subjects; they included 417 (59.48%) females aged 38.45 ± 10.88 years (range 25–65 years) and 284 (40.51%) males aged 42.60 ± 12.03 years (range 25–67 years).

All subjects were physically and mentally healthy, as confirmed by a short unstructured interview. They all provided written informed consent, and the protocol was approved by the Ethics Committee of the Faculty of Medicine, Aristotle University of Thessaloniki, Greece. A detailed description of the study sample and more information can be found elsewhere (43–45).

All data were gathered in the years 2006-2008, before the economic crisis of that period began.

Epidemiological data concerning suicides for the period 2000–2012 were obtained by the Greek National Statistics Authority (www.statistics.gr) and climate data from the Greek National Meteorological Service (http://climatlas.hnms.gr/sdi/). The climate data concerned the season and the year of birth of subjects.

### Methods

The protocol gathered sociodemographic data, including the precise date of birth and parental age at delivery. It also included data from the application of the TEMPS-A and its four temperaments (23, 40–42), the TCI and its seven temperaments and characters and its 25 facets (22, 49–52), and the NEO-PI-3 with its five personality dimensions and their 30 facets (17, 18). The twenty temperament facets of the newly developed cognitive-affective model of temperament (CAMT) were also calculated from the items of the above-mentioned temperament scales (33, 34). In total, 91 variables concerning temperament, character, and personality were used. The Center for Epidemiological Studies-Depression (CES-D) was used for the assessment of depression (53) and the Risk for Assessment of Suicidality Scale (RASS) (48) was used to assess suicidality. Hopelessness was derived from item #8 of the CES-D. All the scales used were already officially validated in the Greek language (43–47, 54).

The dataset included the mean sunlight, mean-minimum, mean-average, and mean-maximum temperatures, as well as the mean precipitation for Greece for the years 1977-2000. These climate data were obtained from the Greek National Meteorological Service and concerned data from the station of Athens (Elliniko) at latitude 37.89°, longitude 23.74° and altitude 47 meters (http://climatlas.hnms.gr/sdi/).

To obtain the season of birth, the seasons were defined in the popular way (March, April, and May for spring, June, July, and August for summer, September, October, and November for autumn and December, January, and February for winter) as well as according to the standard definition of seasons with the utilization of the summer and winter solstices (spring: March 21 to June 20; summer June 21 to September 20; autumn September 21 to December 20, and winter December 21 to March 20).

Zodiacs were defined according to the dates of birth given by the Encyclopedia Britannica (https://www.britannica.com/topic/zodiac).

Also, suicidal data per month were obtained from the Greek National Statistics Authority for the years 2000–2012 and were correlated with the monthly climate data from the years 1977–2000 matched for the birth date of each subject separately. Unfortunately, monthly data for the suicides from 1977–2000 were not available, and monthly climate data for 2000 to 2012 could not be found. The authors considered that, despite climate change, the relative differences between months remain constant, making it fair to correlate climate data from 1977–2000 with suicides from 2000-2012, albeit only as an approximation.

### Statistical analysis

The statistical analysis included the following:

- The creation of descriptive statistics tables concerning age, gender, and the distribution of scale scores in the sample.
- Exploratory factor analysis to search for clustering of months based on climate variables (apart from seasons)
- Analysis of Variance (ANOVA) with Bonferroni correction for multiple comparisons
- Analysis of Covariance (ANCOVA)
- T-test with Bonferroni correction for multiple comparisons
- Pearson correlation coefficient with Bonferroni correction for multiple comparisons
- A conservative and modest Bonferroni correction was applied horizontally by multiplying the original p-values by the arbitrary value of 10. This means that the p-level for significance was set to p<0.005.
- Forward and Backward Stepwise Linear regression analysis (FSLRA and BSLRA).
- The differences observed between males and females made the analysis of the study sample as a whole meaningless; instead it was chosen to analyze separately males and females.

## Results

There were significant differences between males and females concerning several variables after the Bonferroni correction (**Table 1**). Females were younger, had higher depressive and anxious but lower hyperthymic temperaments (from TEMPS-A), higher N (from NEO-PI-3), higher HA, PS, but lower SD (from TCI), higher ER, lower IE, EM, IIE, ECC, and SEI (from CAMT), and higher CES-D and RASS-Life subscales. The two sexes did not differ in terms of parental age or climate variables at birth. Since the two sexes differed in several temperaments, a separate analysis was necessary, and the analysis of the study sample as a whole was considered inappropriate.

**Table 1:** Descriptive statistics of the study variables by sex and comparison between sexes.

	Females		Males		t-value	df	p	
	Mean	SD	Mean	SD				
Age	39.45	10.89	42.60	12.04	-3.605	698	***0.0003***	*
Height (cm)	165.02	5.59	176.88	6.40	-25.650	680	***0.0000***	*
Weight (kg)	66.19	14.15	83.26	13.62	-15.612	671	***0.0000***	*
BMI	24.36	5.49	26.68	3.56	-6.151	667	***0.0000***	*
Paternal age	32.06	6.44	32.06	6.21	0.003	559	0.9975	
Maternal age	26.31	5.47	26.85	6.08	-1.098	556	0.2725	
TEMPS-Depressive	7.99	3.34	6.24	3.10	7.012	699	***0.0000***	*
TEMPS-Cyclothymic	7.34	4.65	6.23	4.31	3.209	699	***0.0014***	*
TEMPS-Hyperthymic	10.92	4.43	12.76	4.37	-5.450	699	***0.0000***	*
TEMPS-Irritable	4.76	3.92	4.48	3.70	0.966	699	0.3345	
TEMPS-Anxious	8.18	5.57	5.05	4.90	7.672	699	***0.0000***	*
NEO-PI-3-N	92.99	18.56	82.37	19.44	7.290	699	***0.0000***	*
NEO-PI-3-E	108.27	16.38	109.76	15.63	-1.205	699	0.2286	
NEO-PI-3-O	105.97	17.49	102.50	16.17	2.663	699	***0.0079***	
NEO-PI-3-A	116.79	16.40	116.07	15.06	0.591	699	0.5545	
NEO-PI-3-C	120.72	19.06	123.88	19.53	-2.133	699	***0.0333***	
TCI-NS	18.87	5.66	19.14	5.86	-0.606	699	0.5447	
TCI-HA	16.14	6.10	13.02	5.51	6.908	699	***0.0000***	*
TCI-RD	14.40	3.85	14.33	3.90	0.213	699	0.8311	
TCI-PS	4.47	1.91	4.44	1.81	0.216	699	0.8291	*
TCI-SD	30.30	6.76	32.83	6.37	-4.987	699	***0.0000***	*
TCI-CO	32.13	5.48	32.04	5.55	0.220	699	0.8260	
TCI-ST	15.92	5.77	14.99	6.17	2.053	699	***0.0405***	
ER	-0.24	0.99	0.38	0.91	-8.432	699	***0.0000***	*
ES	0.01	1.03	0.04	0.97	-0.422	699	0.6731	
SD	0.01	1.04	-0.02	0.98	0.396	699	0.6925	
EB	-0.01	1.06	0.03	0.90	-0.457	699	0.6475	
PSC	-0.01	0.98	0.00	1.04	-0.208	699	0.8349	
EC	0.08	1.05	-0.12	0.94	2.486	699	***0.0132***	
IE	0.13	0.98	-0.15	1.00	3.625	699	***0.0003***	*
EIE	0.07	1.02	-0.12	0.97	2.540	699	***0.0113***	
EM	-0.02	1.00	0.04	1.01	-0.779	699	0.4363	
IIE	-0.12	1.05	0.20	0.91	-4.177	699	***0.0000***	*
IC	-0.11	0.98	0.16	1.01	-3.480	699	***0.0005***	*
EV	-0.02	1.01	0.00	1.00	-0.208	699	0.8355	
SE	-0.02	1.10	0.02	0.85	-0.538	699	0.5910	
EEB	-0.09	1.00	0.14	0.99	-3.087	699	***0.0021***	
EmoS	-0.02	0.95	0.08	1.07	-1.250	699	0.2115	
ECC	-0.15	1.02	0.24	0.94	-5.075	699	***0.0000***	*
CogS	-0.05	1.00	0.06	1.01	-1.541	699	0.1239	
SEB	0.04	1.02	-0.05	0.98	1.097	699	0.2732	
SEI	-0.17	1.00	0.27	0.95	-5.767	699	***0.0000***	*
S	0.00	1.03	0.05	0.97	-0.698	699	0.4856	
CES-D	13.00	9.38	10.07	8.52	4.217	699	***0.0000***	*
Hopelessness CES-D8	1.21	0.97	1.03	0.99	2.338	696	***0.0197***	
RASS-Intention	34.58	88.93	25.74	83.96	1.322	699	0.1868	
RASS-Life	112.13	97.13	86.78	89.49	3.502	699	***0.0005***	*
RASS-History	38.60	46.66	31.44	49.86	1.938	699	0.0530	
Sunlight	233.22	85.03	231.57	87.77	0.249	699	0.8037	
Lowest month temperature	14.11	5.78	14.08	5.84	0.076	699	0.9391	
Mean month temperature	18.50	6.56	18.44	6.65	0.119	699	0.9053	
Max month temperature	22.35	7.10	22.28	7.21	0.141	699	0.8881	
Mean month precipitation	30.40	20.61	30.80	20.94	-0.252	699	0.8015	

*significant after Bonferroni correction.

Significant values are in bold font.

The exploratory factor analysis of climate variables returned a single factor; there were no clear-cut seasons, but variables seem to manifest a bell-shaped curve with a single underlying mechanism. Therefore, in addition to the standard definition of seasons and months used as categorical variables, climate data were also utilized as continuous variables.

The frequency of births did not differ significantly, neither among popularly defined nor standard seasons (for standard seasons, the minimum was observed during autumn at 23.25% and the maximum during summer and spring at 25.67%) nor among months (minimum during October with 7.56% and maximum during August with 10.27%). There were no differences between males and females.

The detailed descriptive data concerning standard seasons are shown in **Table 2**. There were no differences for females among the seasons in any of the tested variables. For males, ANOVAs suggested that there was a difference among seasons after Bonferroni correction concerning paternal (df effect:3, F = 6.077, p=0.0005; higher in winter vs. autumn) but not maternal age (df effect:3, F = 4.038, p=0.008; higher in winter vs. autumn), while IIE was marginal (df effect:3, F = 4.354, p=0.005; lower in autumn and summer vs. spring). ANCOVA with season as the grouping variable, IIE as dependent, and paternal and maternal age as covariates returned significant results for season (SS-8.558, df:3, MS = 2.852, F = 3.633, p=0.013, non-corrected). The complete results, broken down by season and sex are shown in **Table 2**.

**Table 2:** Descriptive statistics of the study variables by standard season.

	Winter					Autumn				Summer				Spring			
	Females		Males			Females		Males		Females		Males		Females		Males	
	Mean	SD	Mean	SD	Mean		SD	Mean	SD	Mean	SD	Mean	SD	Mean	SD	Mean	SD
Age	40.26	10.70	44.24	13.17	38.94		10.69	42.76	11.80	39.17	11.31	40.57	11.89	39.40	10.92	42.94	10.98
Height (cm)	165.18	5.19	176.42	6.07	165.07		5.63	177.42	6.30	165.00	5.68	177.15	6.45	164.85	5.87	176.59	6.88
Weight (kg)	66.24	13.07	81.67	13.52	64.51		10.38	83.14	11.40	67.64	19.59	83.91	16.59	66.24	11.62	84.44	12.33
BMI	24.31	4.80	26.22	3.97	23.70		3.54	26.38	3.11	24.96	7.97	27.13	3.71	24.41	4.47	27.02	3.32
Paternal age	32.54	7.48	***34.25***	***6.57***	31.61		6.22	***29.61***	***5.44***	31.96	6.24	31.97	6.08	32.19	5.97	32.26	5.88
Maternal age	27.00	5.80	***28.57***	***6.37***	25.85		5.39	***24.73***	***5.21***	26.01	4.71	27.17	6.34	26.45	5.87	26.77	5.80
TEMPS-Depressive	8.55	3.45	6.28	3.54	7.19		3.19	5.74	2.83	8.05	3.16	6.36	3.10	8.11	3.42	6.55	2.83
TEMPS-Cyclothymic	7.81	4.97	6.38	4.18	6.39		4.12	5.52	4.38	7.78	4.84	6.12	4.37	7.32	4.56	6.88	4.29
TEMPS-Hyperthymic	10.81	4.16	12.79	4.58	11.26		4.57	12.67	4.41	10.90	4.47	13.17	4.24	10.73	4.56	12.36	4.27
TEMPS-Irritable	4.99	3.91	4.46	3.40	3.97		3.61	4.41	3.80	5.07	4.10	4.74	4.03	4.95	3.99	4.26	3.60
TEMPS-Anxious	8.75	5.79	5.17	5.08	6.85		4.87	4.17	4.14	8.56	6.06	5.28	5.20	8.47	5.35	5.52	5.05
NEO-PI-3-N	95.40	17.63	84.57	20.71	88.24		17.42	79.30	17.72	91.95	20.55	82.00	20.39	95.81	17.73	83.35	18.44
NEO-PI-3-E	106.10	16.64	109.12	14.70	110.16		14.41	111.05	15.35	109.00	17.09	110.29	16.43	107.92	17.00	108.59	16.24
NEO-PI-3-O	103.43	16.58	104.17	16.60	108.00		16.10	100.20	15.88	106.53	19.22	102.49	15.22	106.02	17.73	102.88	17.08
NEO-PI-3-A	114.41	16.45	116.83	14.09	118.74		16.46	115.33	16.78	118.17	16.32	114.33	14.15	115.99	16.28	117.92	15.40
NEO-PI-3-C	119.57	18.29	122.64	19.37	121.13		15.91	126.68	20.21	123.52	21.19	121.41	20.33	118.86	20.06	125.36	17.99
TCI-NS	18.04	5.90	19.54	5.98	19.73		5.08	19.06	5.19	18.23	5.77	19.59	6.02	19.47	5.73	18.24	6.20
TCI-HA	16.98	5.93	13.99	5.94	14.75		5.33	12.18	5.62	15.88	6.44	13.29	5.58	16.82	6.38	12.45	4.67
TCI-RD	13.98	3.64	14.71	3.76	15.43		3.64	14.76	3.53	13.86	3.80	14.51	4.23	14.39	4.12	13.27	3.92
TCI-PS	4.54	1.82	4.39	1.81	4.28		1.88	4.48	1.83	4.67	1.92	4.45	1.81	4.38	1.99	4.42	1.81
TCI-SD	28.94	7.17	32.96	6.05	31.75		6.39	33.23	6.85	30.63	7.03	31.95	7.08	29.98	6.24	33.32	5.33
TCI-CO	32.20	4.74	32.47	5.46	32.42		5.83	31.73	5.77	32.57	5.60	31.55	5.90	31.43	5.68	32.41	5.03
TCI-ST	16.67	5.47	15.12	6.66	14.97		5.72	14.39	5.42	16.58	5.80	14.30	6.01	15.47	5.98	16.21	6.39
ER	-0.36	1.01	0.31	0.90	0.01		0.84	0.52	0.92	-0.24	1.05	0.37	0.96	-0.33	1.00	0.34	0.87
ES	-0.04	1.04	-0.11	0.90	-0.08		0.92	0.26	0.94	0.13	1.02	0.00	1.08	0.01	1.12	0.03	0.94
SD	0.12	0.97	0.01	0.96	-0.01		0.93	-0.02	1.04	0.10	1.13	-0.07	1.01	-0.14	1.10	0.01	0.91
EB	-0.11	1.14	0.09	0.89	0.06		0.99	-0.09	0.92	0.08	1.04	-0.02	0.81	-0.04	1.06	0.15	0.98
PSC	0.18	0.93	0.02	1.14	-0.20		0.97	-0.08	0.95	0.10	1.01	-0.10	0.97	-0.13	0.99	0.19	1.07
EC	-0.09	0.98	-0.01	1.03	0.13		1.04	-0.29	0.93	0.13	1.14	-0.12	0.80	0.12	1.03	-0.05	0.97
IE	0.11	0.99	-0.17	0.91	0.21		0.94	-0.15	0.97	0.19	0.99	-0.25	1.09	0.02	1.01	-0.01	1.03
EIE	0.06	0.90	-0.11	0.90	0.25		1.03	***-0.05***	***1.00***	-0.08	0.97	***-0.05***	***0.98***	0.06	1.14	***-0.30***	***1.01***
EM	-0.04	1.06	0.16	1.05	0.03		0.95	0.05	0.95	-0.07	1.07	0.14	1.02	0.00	0.95	-0.21	0.99
IIE	-0.23	1.18	0.25	0.73	-0.12		1.05	***0.04***	***0.91***	-0.11	1.02	***0.02***	***0.97***	-0.04	0.94	***0.50***	***0.96***
IC	-0.18	1.02	0.10	1.01	-0.04		0.95	0.07	0.95	-0.11	0.98	0.30	0.93	-0.09	0.98	0.16	1.15
EV	-0.10	0.96	0.10	0.96	0.02		1.11	0.02	0.97	-0.11	0.94	-0.06	1.02	0.12	1.01	-0.08	1.06
SE	-0.20	1.17	0.12	0.80	0.19		1.03	-0.08	0.87	-0.09	1.01	-0.04	0.83	0.04	1.14	0.09	0.89
EEB	-0.21	0.99	0.23	1.01	-0.02		0.96	0.12	1.02	-0.07	1.01	0.06	0.96	-0.07	1.04	0.18	1.00
EmoS	-0.06	0.92	-0.06	1.03	0.13		0.84	0.32	1.00	0.06	0.98	0.06	1.22	-0.17	1.00	0.01	0.98
ECC	-0.27	1.05	0.30	0.94	-0.08		0.92	0.25	0.91	-0.19	1.07	0.32	0.99	-0.06	1.03	0.06	0.92
CogS	0.17	0.98	0.08	1.12	-0.22		0.92	0.10	0.91	-0.01	1.00	-0.08	0.93	-0.15	1.06	0.19	1.05
SEB	-0.08	1.01	0.06	0.90	0.21		0.84	-0.10	0.88	-0.03	1.10	0.00	1.05	0.06	1.10	-0.17	1.07
SEI	-0.40	1.09	0.38	0.99	0.00		0.87	0.25	0.95	-0.20	1.01	0.29	0.89	-0.06	0.99	0.14	0.95
S	0.02	1.08	-0.11	0.88	0.02		0.97	0.29	0.89	0.08	0.95	0.13	1.10	-0.12	1.09	-0.09	0.96
CES-D	13.49	9.95	10.92	9.05	12.38		9.32	9.23	7.58	13.14	9.79	10.34	8.60	12.96	8.58	9.61	8.76
Hopelessness CES-D8	1.17	0.97	1.13	0.93	1.09		0.96	1.08	1.07	1.25	0.95	1.00	0.99	1.30	0.99	0.91	0.99
RASS-Intention	30.93	83.55	21.32	94.73	18.56		50.92	21.21	69.09	49.66	112.49	30.72	83.91	37.72	93.21	29.62	85.76
RASS-Life	128.04	97.44	93.55	87.83	86.70		81.24	69.39	70.87	112.79	106.83	94.14	99.78	118.95	97.00	87.88	95.05
RASS-History	42.84	44.95	34.41	50.52	33.76		44.44	30.00	54.20	37.02	50.48	30.86	50.16	40.35	46.55	30.15	44.99

Significant differences are marked with bold italic underlined characters.

Significant values are in bold font.

There was no significant difference among Zodiac signs, even before correction for multiple testing (t-tests in pairs).

There was no correlation among any of the temperament and character variables, depression, hopelessness, and suicidality, and any of the climate variables and parental age for males and females separately. However, in females only, there was a significant positive correlation of weight and BMI with mean sunlight, lowest, mean, and maximum temperatures (all R equal to 0.14), and significant negative correlation with mean precipitation (R=-0.14). Both FSLRA and BSLRA, using BMI and body weight as dependent variables and climate variables as predictors, failed to provide any meaningful models, as the explained variability (R-square) was below 2%.

The Pearson correlation among monthly suicides during 2000–2012 and monthly climate data from 1977–2000 gave significant coefficients for suicides and sunlight (males R = 0.75, females R = 0.42), mean lowest monthly temperature (males R = 0.54, females R = 0.30), mean average monthly temperature (males R = 0.59, females R = 0.32), mean maximum monthly temperature (males R = 0.59, females R = 0.33), and mean precipitation (males R=-0.66, females R=-0.30).

**Figure 1:**
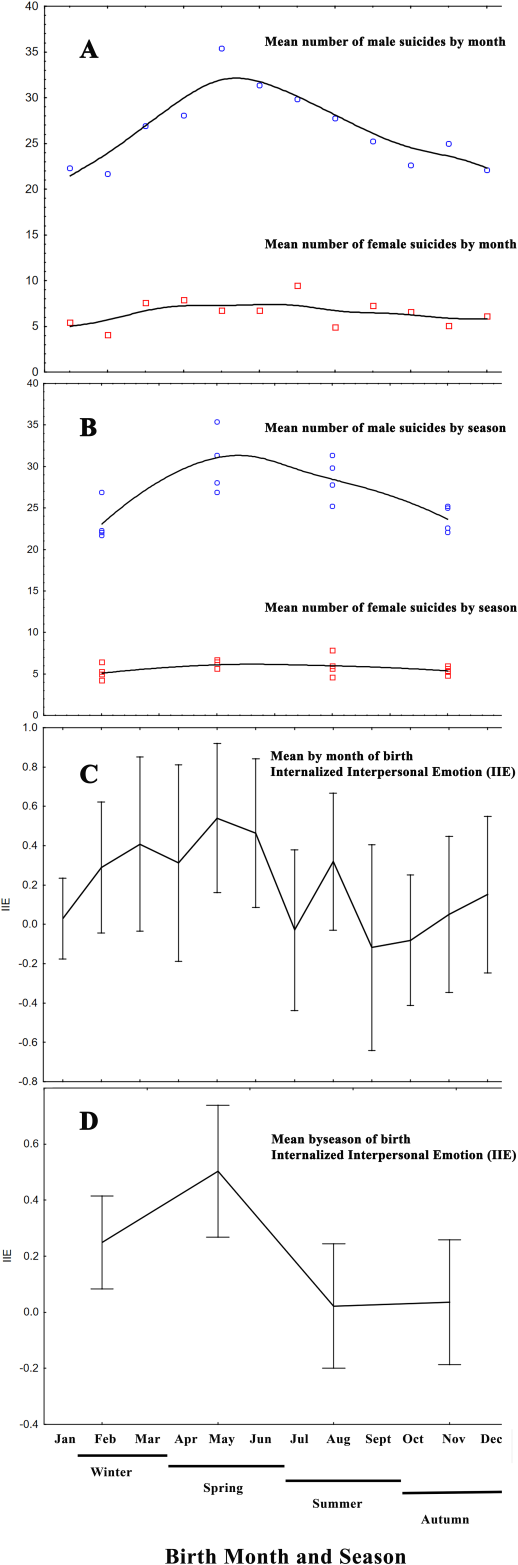
**(A)** Mean number of deaths by suicide for the years 2000–2012 separately in males and females by month of suicide commitment. **(B)** Mean number of deaths by suicide for the years 2000–2012 separately in males and females by season of suicide commitment. **(C)** Mean IEE values by month of birth. **(D)** Mean IEE values by standard season of birth.

The means concerning IEE and male and female suicides by month and season are shown in **Figure 1**. Both IEE and male suicides manifest their peak during the spring, suggesting spring is a risk period both for males to be born and manifest temperamental deficits, and also for committing suicide. Whether these two observations are related cannot be answered by the current study because the data belong to two distinct datasets; however, a hypothesis could be formulated.

## Discussion

The results of the current paper suggest that none of the ‘classic’ temperament and personality variables of the three major models (TEMPS, TCI, and NEO-PI) manifest any relationship with season of birth, climatic variables, or parental age, and this is true even without correction for multiple comparisons. Only the Internalized Interpersonal Emotion (IIE) from the CAMT was related to the season of birth, with high scores for those born during the spring and low scores for those born during the summer and autumn. The finding was significant but relatively marginal and concerned only males. Male deaths by suicide manifested their highest rates during the spring. There was a weak but significant correlation between weight and BMI at present and all climate variables at birth, but, on the contrary, parental age at birth played no role in somatometrics. Overall, in the current study, no role of parental age on the development of temperament and personality was detected, while climate at birth was related only to IEE and BMI.

The Internalized Interpersonal Emotion (IIE) is a temperament dimension introduced recently in the frame of a new cognitive-affective model of temperament, the CAMT (33, 34). It includes concepts similar to the TCI facets of attachment: intimacy vs. privacy (expression of experiences and feelings, warm and lasting social attachment, sensitivity to rejection and slights) and purposefulness vs. lack of goal direction (delay gratification to achieve goals vs. reactiveness and empty lives). High scores of IEE could be interpreted as corresponding to poor emotional and social life, as well as to relationships with others, and problematic interpersonal attachment. These high scores (and problematic interpersonal relationship) were observed in those born during the spring.

The relationship of problematic attachment with suicidality has been reported previously, especially as a problematic area during the recent COVID-19 pandemic (55–59),

There are only a few studies that have reported on the effect of the season of birth on the development of temperament in adult life. However, results vary depending on the theory and the scale used for the approach. Those studies that used the TCI support the manifestation of lower Novelty Seeking (NS) in those females born during the winter. Interestingly, this does not hold true for adolescent girls, for whom the association is reverse, suggesting that being born during the winter might define a specific developmental trajectory for females only (60–62). The same group of researchers reported inconclusive results for Reward Dependence (RD), with one of their studies (from Finland) reporting that males born during spring manifested significantly lower Reward Dependence (RD) scores in comparison to those born during the autumn, while a second one (from Sweden) reported that RD scores manifested their highest values for both genders born in December (60, 61). Other researchers reported that Disorderliness (NS4) was higher for males born in autumn, and Extravagance (NS3) was high for males born in summer and winter (63). The studies that utilized the NEO-PI big-five personality traits reported that winter-born individuals manifested lower Agreeableness scores (60), summer-born males manifested lower Conscientiousness scores (61), and those persons born during the cold months manifested higher Extraversion scores (64, 65). Interestingly, the results for Neuroticism are inconsistent, with those born during the cold months manifesting lower scores (65), and those born during the summer higher scores (66). However, a third study reported higher scores in winter-born (64). The use of the TEMPS-A model mainly returned negative results after correction (67, 68). One study with a different approach reported that schizotypy is higher in those born in winter (69). In contrast, another one reported that the season of birth may determine the development of emotional and behavioral regulation skills during early infancy (10). Our findings do not align directly with any of the above, but they may concur in that a common underlying factor among the results is disordered affectivity in interpersonal relationships, a notion strongly supported by our findings.

There are no hard data on the possible explanation and the neurobiological background that could exist behind the correlation of environmental variables and especially of climate with temperament and personality. Therefore all theories should be considered as purely speculative. The explanations include that these effects could be mediated by vitamin D in the mother, which has been linked to negative affectivity in the offspring (70), or it could be the result of a variation in monoamine metabolism (71, 72). At the same time, differences in the size of specific brain areas have been reported, with higher volumes for those born during the winter, and this has been found with the use of MRI (73). It has been specifically reported that the season of birth exerts a modifying effect on the relationship between the Brain-Derived Neurotrophic Factor (BDNF) and Harm Avoidance, but whether this is a direct or a triangular relationship remains to be shown (74).

Concerning suicidality, the season of birth seems to be an independent determinant (12) with a higher risk of death for those born during spring-summer (11, 75–80) as well as for the choice of more potentially lethal methods (12). Birth during the autumn has been reported to constitute a risk for adolescent self-mutilative behavior (81). Interestingly, modern lifestyle tends to minimize the effect of birth season on suicidality, probably because of the increasingly modifying effect of modern life on the living environment and its micro-climate (82).

Also, the climate seems to play a role, with high lethality of attempts observed during periods of high sunshine (83, 84), high temperature (84–91), and low humidity (86). Age at onset and suicidality in bipolar patients have been related to large changes in sunshine from winter to summer (92–97). However, the seasonality of suicidality might not be related to climate per se (80, 98).

Our findings do not support a relationship between the season of birth and current suicidal thoughts in the adult general population. However, previous studies from our group suggested that periods of high or low temperature could be related to higher rates of both attempted and completed suicide (67, 98–100), and this is in accord with the data from the Greek Statistics Authority that are reported here. Overall, our results suggest that spring could be related to deficits in affective interpersonal relating for those born during that season, and also to higher deaths by suicide in adults again during the same season. This could mean that this period of the year exerts an adverse effect on mental health, at least on vulnerable populations.

The finding that, in females only, there was a positive correlation of weight and BMI with mean sunlight, lowest, average, and maximum temperature (all R equal to 0.14), and negative with mean precipitation (R=-0.14) is challenging to interpret. Whether this is a direct relationship or it is mediated by temperament and personality is impossible to answer. The first to suggest that body type is related to personality and mental disorders were Ernst Kretschmer (1888–1964) and William H. Sheldon (1899–1977). They proposed that there is an association between specific body types, personality traits, and mental disorders and described four body types: the pyknic, the athletic, the asthenic, and the dysplastic. Their theories are not of academic value anymore, but continue to influence lay people (101–105). In the general population, higher BMI is related to somatic/vegetative symptoms and cognitive symptoms of depression, but not to mood per se (106), although data suggest this relates to atypical depressive symptoms (107). A systematic review reported that the season of birth is associated both with high birth weight and higher BMI in childhood for those born during the winter (7). At the same time, a large-scale study confirmed these findings only concerning birth weight (108).

While most theories imply a role for vitamin D, in terms of psychoendocrinology, the hormone ghrelin, but not leptin, is associated with BMI and depressive status (109, 110). There are also genetic studies reporting a common genetic background between early stress, high BMI, and schizophrenia, bipolar disorder, and unipolar depression. At the genetic level, an extensive polygenic overlap between BMI and schizophrenia, bipolar disorder, and unipolar depression has been reported (104). Interestingly, higher BMI polygenic scores correlate with the presence of higher early life stress, which in turn predicted higher current depressive symptoms (105). In adult depressive patients, cross-sectionally, there is a strong correlation between BMI and depressive symptoms in both sexes (111, 112), and this relationship seems to be stronger in females (113, 114), which is in accord with our findings.

Concerning the relationship between season of birth and adult BMI, a working hypothesis could implicate the hypothalamus and more specifically the central melanocortin system, which acts through melanocortin 3 and 4 receptors (MC3R and MC4R) to control appetite, food intake, and energy expenditure, and it is directly related to body development (115, 116). Whether mutations in the genes controlling the structure of these receptors (113, 114) or impaired input, training/regulation of their function by environmental factors during early life could be responsible, should be the focus of targeted research (117).

### Strengths of the current study

1. The current study utilized a large, adequately sized sample of normal individuals, more or less representative of the country’s healthy and active population, and is equivalent in size and quality to the samples of previous similar studies.
2. The use of the three major questionnaires of temperament and character makes this model development unique in the literature.

### Limitations of the current study

1. Limitations of the linear methods: The methods utilized in the current study were linear and of first-order. The aim was to search for basic and fundamental relationships but a higher-order non-lineal model cannot be excluded.
2. Limitations because of the questionnaires included: The scales included in the current study reflect aspects of temperament, character, and personality but they do not reflect all theoretical or empirical approaches. There is the possibility the findings were biased toward the theories underlying these questionnaires rather than true psychological structure per se.
3. Limitation because of confounding variables: The presence of confounding variables and triangular correlations are standard problems in psychiatric research. Especially specific sociocultural and socioeconomic variables related to climate and to birth could not be excluded.
4. Future research should focus on neurobiological differences in relationship with the season of birth and climate, especially in the face of the changes in mental health the climate change might cause. In this way the input will not be restricted to inner experience as it is perceived and described consciously by the individual.

## Data Availability

The original contributions presented in the study are included in the article/Supplementary Material. Further inquiries can be directed to the corresponding author.
